# Estimation of potato leaf area index based on spectral information and Haralick textures from UAV hyperspectral images

**DOI:** 10.3389/fpls.2024.1492372

**Published:** 2024-11-22

**Authors:** Jiejie Fan, Yang Liu, Yiguang Fan, Yihan Yao, Riqiang Chen, Mingbo Bian, Yanpeng Ma, Huifang Wang, Haikuan Feng

**Affiliations:** ^1^ Key Laboratory of Quantitative Remote Sensing in Agriculture of Ministry of Agriculture and Rural Affairs, Information Technology Research Center, Beijing Academy of Agriculture and Forestry Sciences, Beijing, China; ^2^ College of Geomatics, Xi’an University of Science and Technology, Xi’an, China; ^3^ Key Lab of Smart Agriculture System, Ministry of Education, China Agricultural University, Beijing, China; ^4^ College of Information and Management Science, Henan Agricultural University, Zhengzhou, China; ^5^ Ecological Meteorology and Satellite Remote Sensing, Beijing Municipal Climate Center, Beijing, China; ^6^ College of Agriculture, Nanjing Agricultural University, Nanjing, China; ^7^ Collaborative Innovation Center for Modern Crop Production Co-sponsored by Province and Ministry, Nanjing Agricultural University, Nanjing, Jiangsu, China

**Keywords:** potato, Unmanned Aerial Vehicle, hyperspectral, Haralick textures, leaf area index

## Abstract

The Leaf Area Index (LAI) is a crucial parameter for evaluating crop growth and informing fertilization management in agricultural fields. Compared to traditional methods, UAV-based hyperspectral imaging technology offers significant advantages for non-destructive, rapid monitoring of crop LAI by simultaneously capturing both spectral information and two-dimensional images of the crop canopy, which reflect changes in its structure. While numerous studies have demonstrated that various texture features, such as the Gray-Level Co-occurrence Matrix (GLCM), can be used independently or in combination with crop canopy spectral data for LAI estimation, limited research exists on the application of Haralick textures for evaluating crop LAI across multiple growth stages. In this study, experiments were conducted on two early-maturing potato varieties, subjected to different treatments (e.g., planting density and nitrogen levels) at the Xiaotangshan base in Beijing, during three key growth stages. Crop canopy spectral reflectance and Haralick textures were extracted from ultra-low-altitude UAV hyperspectral imagery, while LAI was measured using ground-based methods. Three types of spectral data—original spectral reflectance (OSR), first-order differential spectral reflectance (FDSR), and vegetation indices (VIs)—along with three types of Haralick textures—simple, advanced, and higher-order—were analyzed for their correlation with LAI across multiple growth stages. A model for LAI estimation in potato at multiple growth stages based on spectral and textural features screened by the successive projection algorithm (SPA) was constructed using partial least squares regression (PLSR), random forest regression (RFR) and gaussian process regression (GPR) machine learning methods. The results indicated that: (1) Spectral data demonstrate greater sensitivity to LAI than Haralick textures, with sensitivity decreasing in the order of VIs, FDSR and OSR; (2) spectral data alone provide more accurate LAI estimates than Haralick textures, with VIs achieving an accuracy of R² = 0.63, RMSE = 0.38, NRMSE = 28.36%; and (3) although Haralick textures alone were not effective for LAI estimation, they can enhance LAI prediction when combined with spectral data, with the GPR method achieving *R*² = 0.70, RMSE = 0.30, NRMSE = 20.28%. These findings offer a valuable reference for large-scale, accurate monitoring of potato LAI.

## 1 Introduction

The potato, ranked fourth in the global list of food crops, is irreplaceable in achieving development goals such as the eradication of hunger, poverty and malnutrition (Ahmed et al., 2024). Its production in developing countries has grown significantly over the past two decades, making the monitoring of its growth critical to the security and stability of the global food supply (Badr et al., 2022). The Leaf Area Index (LAI) represents the total one-sided leaf area of a crop per unit ground area and is closely linked to key physiological processes such as respiration, transpiration, productivity, and photosynthesis (Li et al., 2016; Ilniyaz et al., 2022). Rapid and accurate estimation of LAI is essential for diagnosing crop growth and predicting yield (Cohrs et al., 2020; Kaplan and Rozenstein, 2021). While traditional manual measurement methods are precise, they are also time-consuming, destructive to crops, and incapable of providing large-scale spatiotemporal data, thereby limiting the effectiveness of crop management. Remote sensing, which involves detecting and obtaining information about objects from a distance without direct contact (Kokubu et al., 2020; Raj et al., 2021), has become increasingly important in monitoring crop LAI due to its non-destructive nature and high-throughput capabilities. Among the various remote sensing technologies available, UAV (Unmanned Aerial Vehicle) remote sensing has emerged as the preferred method for estimating crop LAI, owing to its ability to configure multiple sensors, its flexibility in data collection, and the high spatial and temporal resolution of the images it captures (Dong et al., 2019; Revill et al., 2020).

There are three primary approaches to crop LAI estimation using UAV remote sensing imagery: physical models, hybrid models, and empirical statistical models (Verrelst et al., 2015). Physical models are based on light-crop interactions, such as reflection and absorption, providing a mechanistic and generalizable framework (Fu et al., 2022). These models simulate crop canopy spectral reflectance, which can indirectly estimate LAI. However, despite their mechanistic nature, physical models require numerous input parameters, making them costly to implement. Hybrid models leverage simulation data from physical models to train machine learning algorithms, offering an effective method for estimating LAI without extensive ground-truthing data (Verrelst et al., 2015). On the other hand, empirical statistical models establish a statistical relationship between crop characteristics and LAI (Cao et al., 2020). This approach is user-friendly, easy to implement, and has gained popularity. UAV hyperspectral remote sensing has high spectral resolution and strong band continuity, and its original spectral reflectance (OSR) contains a large amount of spectral information, which can be effectively used for crop physicochemical parameter estimation (Chen et al., 2024). In order to improve the accuracy of hyperspectral remote sensing in the estimation of crop physicochemical parameters, the OSR need to be preprocessed to reduce noise and improve accuracy. First-order differential spectral reflectance (FDSR) not only eliminates the effect of background, but also resolves the overlapping signals and improves the sensitivity of spectral features to crop physicochemical parameters. Additionally, vegetation indices (VIs) are also an effective form of extracting spectral features of crop canopies, which are mainly formed by combinations of reflectance in two or more bands in the visible to near-infrared region (Fan et al., 2022; Liu et al., 2024b). Optical VIs can mitigate the effects of canopy structure and soil on crop reflectance spectra, thereby enhancing and highlighting relevant crop information (Liu et al., 2024a). Numerous VIs are currently used to estimate crop LAI. For example, Lu et al. (2022) evaluated the performance of several VIs in estimating wheat LAI at multiple growth stages and found that the normalized red-edge index exhibited a significant linear relationship with LAI (R² = 0.53). Similarly, Gong et al. (2021) assessed the LAI of rice throughout its growth stages using different VIs and discovered that the Enhanced Vegetation Index 2 produced higher estimation accuracy (R² = 0.38). These studies show a gradual decrease in the sensitivity of VIs to LAI over different growth stages, thus challenging the use of VIs for multiple growth stages LAI estimation.

The band reflectance used to form VIs is primarily influenced by leaf morphology and canopy structure (Croft et al., 2014; Zhang et al., 2021b). Compared to maize and wheat, potatoes exhibit more complex changes in canopy structure throughout their growth phases (Li et al., 2020; Yang et al., 2021). During the nutrient reserve stage, potato stems grow randomly, and leaves increase in size. As the crop enters the pre-mid reproductive stage, the canopy becomes denser, leading to VI saturation under high LAI conditions. As underground tubers begin to form, nutrients accumulated during the earlier growth stages are transferred to these tubers. At this stage, potato leaves start to yellow and wither, significantly affecting the reflectance spectrum of the potato canopy. Consequently, estimating LAI without considering the growth stage of the potato, which accounts for changes in canopy structure, is challenging. To achieve accurate LAI estimation, the differences in potato canopy structure across growth stages must be quantified. Texture features of an image, which define the variability between neighboring pixels within a given window, can describe variations in canopy structure (Wulder et al., 1998; Yue et al., 2019; Zhang et al., 2022c). Recent studies have incorporated texture features when estimating LAI across multiple crop growth stages (Li et al., 2019). For instance, Qiao et al. (2024) improved multi-growth stage LAI estimation in peanut by combining VIs with image texture features extracted using the Gray-Level Co-occurrence Matrix (GLCM). Similarly, Zhou et al. (2022) demonstrated that texture features extracted using discrete wavelet transform, when combined with VIs, provided more accurate LAI estimates for rice compared to using VIs alone. While combining texture features with VIs is expected to enhance LAI estimation across different crop growth stages, it remains uncertain whether existing texture feature extraction methods can accurately capture the complex changes in potato canopy structure at various growth stages. Therefore, it is essential to explore texture features that effectively reflect these structural changes to assist VIs in improving LAI estimation accuracy across multiple growth stages in potatoes.

Among texture features, the Haralick texture feature is the most prominent, relying on the GLCM. Typically, one or more summary statistics, known as Haralick features, are used to summarize the GLCM for ease of interpretation (Haralick et al., 1973; Das and Naskar, 2024). Different types of texture features, including simple, advanced, and high-order features, can be extracted using the Haralick texture extraction method based on the GLCM (Jiang et al., 2021). Among these features, it is possible to filter out those that are particularly sensitive to changes in potato canopy structure, which, when combined with VIs, can enable accurate estimation of LAI across multiple growth stages. Compared to digital or multispectral images, UAV hyperspectral images can provide richer spectral information. When captured under ultra-low-altitude flight conditions, these high-spatial-resolution hyperspectral images can also extract canopy texture features that respond to crop LAI (Duan et al., 2019). Consequently, UAV-based hyperspectral imaging technology offers significant potential for improving LAI estimation. However, extracting sensitive features from hyperspectral image-derived variables is challenging due to the high dimensionality and complexity of the data. As a result, sensitive feature selection methods, such as genetic algorithms, successive projection algorithms (SPA), and stepwise regression algorithms, have been employed to identify the optimal combination of spectral and texture features (Gu et al., 2022; Ji et al., 2022; Ma et al., 2022). Zhang et al. (2021a) demonstrated that features extracted using SPA combined with machine learning can yield more accurate LAI estimation results. Although machine learning methods are increasingly powerful tools for analyzing spatial variations in LAI from multiple remotely sensed feature datasets, not all machine learning approaches are equally effective for crop LAI estimation at multiple growth stages. The choice of appropriate regression methods is also crucial for improving the accuracy of LAI estimation. To the best of our knowledge, few studies have investigated the use of hyperspectral imagery to extract spectral and Haralick texture features for estimating LAI across multiple growth stages in potato.

To enhance the accuracy of potato LAI estimation across multiple growth stages, this study aimed to: (1) evaluate the performance of spectral information and Haralick texture features extracted from UAV hyperspectral imagery for LAI estimation independently; (2) determine whether combining spectral information with Haralick texture features improves LAI estimation accuracy; and (3) identify the optimal combination of variables and the most effective regression method for estimating potato LAI at various growth stages. The findings of this study offer insights into utilizing UAV hyperspectral imaging technology to obtain both canopy spectral and structural information, thereby improving the precision of LAI estimation and establishing a foundation for monitoring potato growth at the field scale.

## 2 Materials and methods

### 2.1 Experiment design

**Figure 1:**
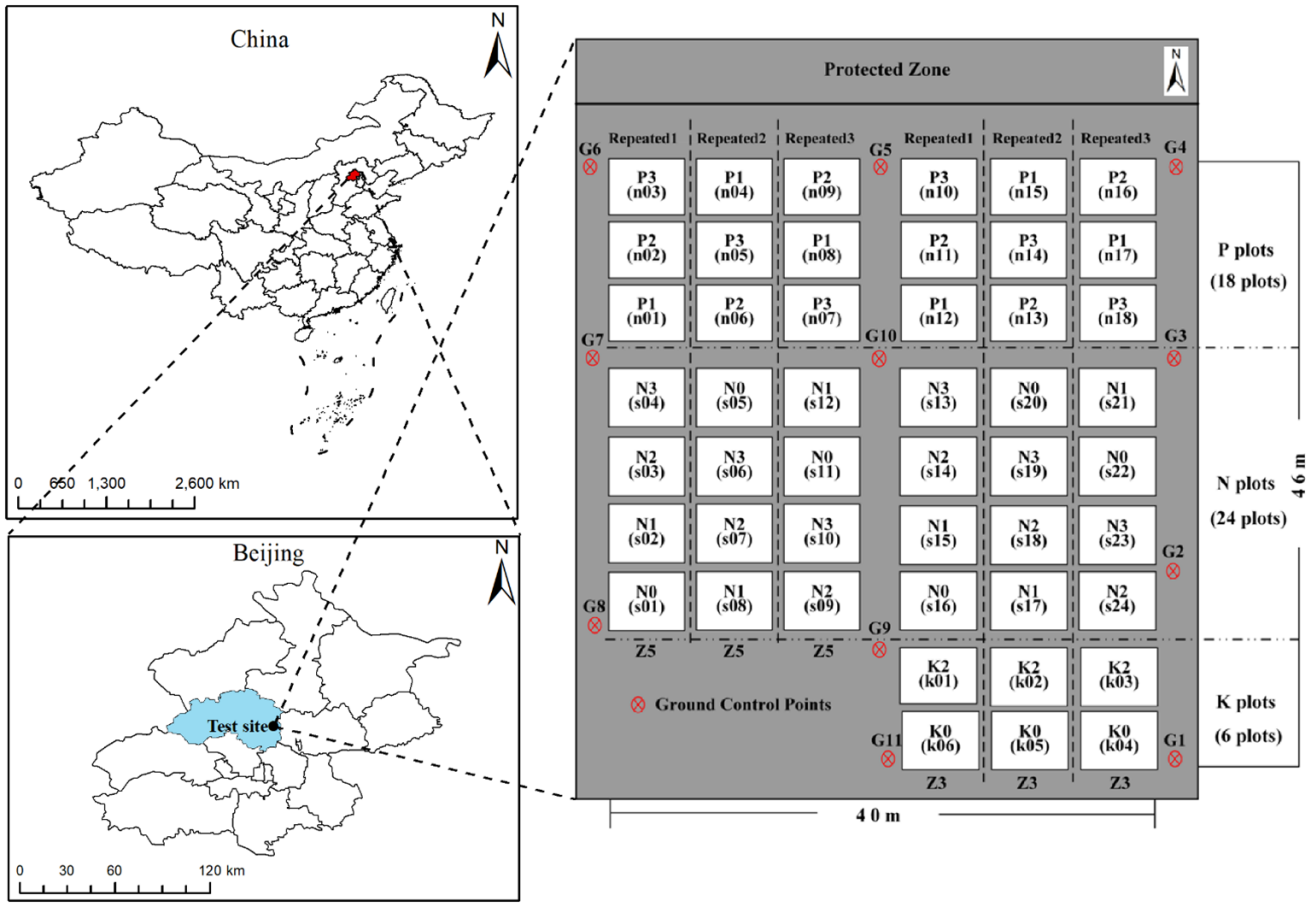
The spatial planting distribution of potatoes in this study.

Potato trials were conducted at the National Precision Agriculture Experimental Base in Changping District, Beijing, China (**Figure 1**). Two early-maturing potato varieties, Zhongshu 3 (Z3) and Zhongshu 5 (Z5), were planted. To increase the spatial variability of LAI, three treatments were performed, including different planting densities, nitrogen gradients, and potassium gradients. Planting density was categorized into three levels: 60,000 (P1), 72,000 (P2), and 84,000 (P3) tubers/hm^2^. Nitrogen treatments were categorized into four levels: 0 (N0), 112.5 (N1), 225 (N2), and 337.5 (N3) kg/hm^2^ of pure nitrogen. Potash treatments were categorized into three levels: 0 (K0), 495 (K1, applied to the planting density and nitrogen test areas), and 990 (K2) kg/hm^2^ of K_2_O. In this experiment, each plot size of 32.5 m^2^ was applied with 90 kg/hm2 P2O5 and each treatment was replicated three times, totalling 48 plots. Field management practices, including weeding, fertilization, and irrigation, were consistent with local agricultural conditions. To calibrate the UAV hyperspectral images acquired during the study, 11 ground control points were evenly distributed around the experimental field. The spatial locations of these points were determined using the Qianxun SR2 high-precision RTK measurement system.

### 2.2 Hyperspectral images acquisition and processing

In 2019, UAV hyperspectral images were acquired during three critical stages of potato development: tuber formation (28 May), tuber growth (10 June), and starch accumulation (20 June). These stages correspond to BBCH codes 41, 44, and 47 (referred to as BBCH41, BBCH44, and BBCH47), respectively. The images were captured using an M600 UAV (SZ DJI Technology Co., Ltd., Shenzhen, China) equipped with a UHD 185-Firefly imaging spectrometer (Cubert GmbH, Ulm, Germany). The UHD185 has 125 spectral channels covering a wavelength range of 450–950 nm, with a sampling interval of 4 nm. The UAV followed identical flight paths across all three growth stages, maintaining consistent take-off positions. The image acquisition was conducted under clear, cloudless, and windless conditions, between 11:30 am and 13:30 pm, when direct sunlight reached the ground. Flights were carried out at an altitude of 20 meters, resulting in a spatial resolution of 1.43 cm, with a flight speed of 1.5 m/s. To facilitate the efficient stitching of orthophotos, the heading and sidelap overlaps were manually set to 85% and 93%, respectively. Given the small size of the field, each hyperspectral image acquisition took approximately 12 minutes. Before each take-off, dark current and radiometric calibration were performed on the ground using a black-and-white calibration plate.

The processing of UAV hyperspectral images involves several key steps: panchromatic image stitching, fusion of panchromatic and hyperspectral images, and extraction of canopy spectral reflectance. First, Agisoft PhotoScan software (Agisoft LLC, St. Petersburg, Russia) was used to correct the topography of the panchromatic images by integrating the positional data from the ground control points. Next, the panchromatic images were mosaicked using the structure-from-motion algorithm, and orthophotos of the test field were generated. The panchromatic orthophoto was then fused with the hyperspectral image using Cube-Pilot software (Cubert GmbH, Ulm, Germany) to create a new hyperspectral image. Finally, the average spectral reflectance of the potato canopy was extracted from the vector files of the 48 plots using ENVI software, and this data was used for subsequent analysis.

### 2.3 LAI measurement

To obtain LAI data, three uniformly growing plants were destructively sampled at each growth stage of tuber formation, tuber growth, and starch accumulation. Plants were transported quickly to the laboratory to prevent water loss leading to leaf curling. After manual separation of the stems and leaves, 60 leaf discs were punched out with a 0.8 cm diameter punch and their wet weights were measured using a high-precision balance (W1). The remaining leaves of the three plants were also measured for their wet weight (W2). Finally, the LAI of each plot was calculated using the weight ratio and planting density. The formula for LAI was as follows:


(1) $$LAI=\frac{\left(W1+W2\right)}{3\times W1}\times S\times M$$

Where WI and W2 were the wet weight of 60 leaf discs and the remaining leaves of three plants respectively. S was the area of leaf discs. M was the number of potato plants per unit area.

### 2.4 The extraction of spectral and textural feature

The spectral information extracted from UAV hyperspectral images in this study primarily includes canopy original spectral reflectance (OSR), first-order differential spectral reflectance (FDSR), and vegetation indices (VIs). Additionally, simple, advanced, and higher-order texture features were derived using Haralick texture extraction methods.

#### 2.4.1 The extraction of FDSR

A first-order differential transformation of the raw canopy spectra can eliminate the interference of linear background noise on the canopy spectral signals and enhance the spectral absorption and reflectance features in the visible to near-infrared region, which is conducive to the improvement of the spectral reflectance response to LAI (Imanishi et al., 2004; Li et al., 2014). Hyperspectral data are well-suited for first-order differential processing due to their large number of continuous bands. The first-order differentiation formula used in this study is as follows:


(2) $${\text{FDSR}}_{\lambda \left(i\right)}=\frac{R_{\lambda \left(i-1\right)}-R_{\lambda \left(i+1\right)}}{\text{Δ}\lambda }\;$$

where ${\text{FDSR}}_{\lambda \left(i\right)}$ is the first-order differential spectral reflectance at a central wavelength i between waveband i−1 and i+1 . $R_{\lambda \left(i-1\right)}$ and $R_{\lambda \left(i+1\right)}$ are the reflectance in the waveband i−1 and i+1 , respectively. Δλ is the sampling interval.

#### 2.4.2 The extraction of VIs

VIs are effective spectral signals used to enhance vegetation information (Sone et al., 2009; Hu et al., 2021; Wengert et al., 2021). In this study, thirty VIs were selected for estimating LAI, with their specific expressions and names provided in **Table 1**.

**Table 1:** The VIs used in the study.

VIs	Number	Formula	Reference
Difference vegetation index (DVI)	1	*R*_890_-*R*_670_	(Richardson and Wiegand, 1977)
Enhanced vegetation index (EVI)	2	2.5×(*R*_800_-*R*_670_)/(*R*_800_+6×*R*_670_-7.5×*R*_450_+1)	(Ahamed et al., 2011)
Enhanced vegetation index2 (EVI2)	3	2.5×(*R*_800_-R_670_)/(*R*_800_+2.4×*R*_670_+1)	(Jiang et al., 2008)
Green normalized-difference vegetation index (GNDVI)	4	(*R*_750_-*R*_550_)/(*R*_750_+*R*_550_)	(Gitelson et al., 1996)
Greenness index (GI)	5	*R*_554_/*R*6_77_	(Zarco-Tejada et al., 2005)
Linear combination index (LCI)	6	(*R*_850_-*R*_710_)/(*R*_850_+*R*_670_)^1/2^	(Datt, 1999)
Modified chlorophyll-absorption ratio index (MCARI)	7	((*R*_700_-*R*_670_)-0.2×(*R*_700_-*R*_550_))(*R*_700_/*R*_670_)	(Haboudane et al., 2002)
Modified simple ratio index (MSR)	8	(*R*_800_/*R*_670_-1)/(*R*_800_/*R*_670_+1)^1/2^	(Chen, 1996)
Modified soil adjusted vegetation index (MSAVI)	9	0.5×[2×*R*_800_+1−((2×*R*_800_+1)^2^-8×(*R*_800_-*R*_670_))^1/2^]	(Haboudane et al., 2002)
Modified triangular vegetation index 1 (MTVI1)	10	1.2×[1.2×(*R*_800_-*R*_550_)-2.5(*R*_670_-*R*_550_)]	(Haboudane et al., 2004)
Modified triangular vegetation index 2 (MTVI2)	11	1.5×(1.2×(*R*_800_-*R*_500_)-2.5×(*R*_670_-*R*_550_))/ (2×(*R*_800_+1)^2^-(6×*R*_800_-5×(*R*_670_)^1/2^)-0.5)^1/2^	(Haboudane et al., 2004)
Normalized-difference vegetation index (NDVI)	12	(*R*_800_-*R*_680_)/(*R*_800_+*R*_680_)	(Rouse et al., 1974)
Normalized difference red edge (NDRE)	13	(*R*_790_-*R*_720_)/(*R*_790_+*R*_720_)	(Fitzgerald et al., 2010)
Normalized pigment chlorophyll ratio index (NPCI)	14	(*R*_670_-*R*_460_)/(*R*_670_+*R*_460_)	(Peñuelas et al., 1994)
Normalized difference index (NDI)	15	(*R*_850_-*R*_710_)/(*R*_850_+*R*_680_)	(Apan et al., 2003)
Nitrogen reflectance index (NRI)	16	(*R*_570_-*R*_670_)/(*R*_570_+*R*_670_)	(Barnes et al., 1992)
Optimizing soil-adjusted vegetation index) (OSAVI)	17	1.16×(*R*_800_-*R*_670_)/(*R*_800_+*R*_670_+0.16)	(Li et al., 2016)
Plant senescence reflectance index (PSRI)	18	(*R*_680_-*R*_500_)/*R*_750_	(Merzlyak et al., 1999)
Pigment‐specific normalized difference (PSND)	19	(*R*_800_-*R*_470_)/(*R*_800_+*R*_470_)	(Blackburn, 1998)
Plant biochemical index (PBI)	20	*R*_810_/*R*_560_	(Rama Rao et al., 2008)
Ratio vegetation index (RVI)	21	*R*_810_/*R*_660_	(Xue et al., 2004)
Renormalized-difference vegetation index (RDVI)	22	(*R*_800_-*R*_670_)/(*R*_800_+*R*_670_)^1/2^	(Roujean and Breon, 1995)
Ratio analysis of reflectance spectra (RASI)	23	*R*_760_/*R*_500_	(Chappelle et al., 1990)
Red‐edge vegetation stress index (RVSI)	24	[(*R*_712_+*R*_752_)/2]-*R*_732_	(Merton and Huntington, 1999)
Soil-adjusted vegetation index (SAVI)	25	1.5×(*R*_800_-*R*_670_)/(*R*_800_+*R*_670_+0.5)	(Huete, 1988)
Spectral-polygon vegetation index (SPVI)	26	0.4×[3.7×(*R*_800_-*R*_670_)-1.2×|*R*_550_-*R*_670_|]	(Vincini et al., 2006)
Triangular vegetation index (TVI)	27	0.5×[120×(*R*_750_-*R*_550_)-200×(*R*_670_-*R*_550_)]	(Broge and Leblanc, 2001)
Transformed chlorophyll absorption ratio index (TCARI)	28	3×[(*R*_710_-*R*_680_)-0.2×(*R*_700_-*R*_560_)(*R*_710_/*R*_680_)]	(Haboudane et al., 2002)
Visible atmospherically resistance index (VARI)	29	(*R*_555_-*R*_680_)/(*R*_555_+*R*_680_-*R*_480_)	(Gitelson et al., 2003)
Wodified wide dynamic range vegetation index (WDRVI)	30	(0.1×*R*_800_-*R*_670_)/(0.1×*R*_800_+*R*_670_)	(Stamatiadis et al., 2006)

#### 2.4.3 The extraction of Haralick texture features

Haralick texture features are computed from a GLCM, which records the co-occurrence of neighboring gray levels in an image (Brynolfsson et al., 2017). The GLCM is a square matrix, where the number of rows and columns corresponds to the number of gray levels in the region of interest. Each texture feature is derived from the elements of the GLCM and represents a specific relationship between neighboring voxels. In this study, we utilized the Python package and Orfeo Toolbox 7.2.0 (https://www.orfeo-toolbox.org/download/) to extract the Haralick features.

In order to reduce the difficulty of extracting texture features, principal component analysis (PCA) was performed on the raw hyperspectral images using ENVI software. It was found that the first principal component image could explain 90% of the image information. Consequently, various Haralick texture features were extracted from this first principal component image. Simple, advanced, and higher-order textures were derived using a 5×5 window size in the 45° direction. The names and quantities of the extracted Haralick texture features are listed in **Table 2**.

**Table 2:** The Haralick texture used in the study.

Type of textures	Number	Names
simple	1	Energy
	2	Entropy
	3	Correlation
	4	Inverse difference moment
	5	Inertia
	6	Cluster shade
	7	Cluster prominence
	8	Haralick correlation
advanced	9	Mean
	10	Sum of squares: variance
	11	Dissimilarity
	12	Sum average
	13	Sum variance
	14	Sum entropy
	15	Difference variance
	16	Difference entropy
	17	Information measures of correlation IC1
	18	Information measures of correlation IC2
higher order	19	Short run emphasis
	20	Long run emphasis
	21	Grey-level nonuniformity
	22	Run length nonuniformity
	23	Run percentage
	24	Low grey-Level run emphasis
	25	High grey-Level run emphasis
	26	Short run low grey-level emphasis
	27	Short run high grey-level emphasis
	28	Long run low grey-level emphasis

### 2.5 Statistical analysis and methodology

The optimal combination of variables for estimating LAI was determined using the SPA. SPA is a forward variable selection algorithm based on the projection analysis of vectors (Sun et al., 2019). Initially, a set of vectors is randomly selected as the starting variables. These vectors are then projected onto the unselected vectors, and the magnitudes of the projected vectors are compared. The variable with the largest projected vector is selected, and this process is repeated through several iterations. Ultimately, the optimal combination of variables is chosen based on the minimum root mean square error (RMSE) of cross-validation.

Partial Least Squares Regression (PLSR) is a statistical method used to develop a linear regression model by projecting independent and dependent variables into a new space. This method involves both the extraction of principal components from the independent and dependent variables and the maximization of the correlation between these components during the regression modeling process (Tao et al., 2020). Random Forest Regression (RFR) is a machine learning technique that integrates a large number of decision trees (Yue et al., 2018). The accuracy of the RFR model is primarily influenced by the number of decision trees (ntree) and the number of nodes (mtry), with mtry typically set to one-third of the input variables and ntree set to 500. Gaussian Process Regression (GPR) is a method based on Bayes’ theorem that models the relationship between dependent and independent variables using a kernel function (Liu et al., 2022b). Compared to other popular machine learning methods, GPR models are simpler to optimize and particularly suitable for training on small sample datasets. In this study, MATLAB software was used for training and validating LAI models with the aforementioned machine learning methods. A total of 96 samples (replicates 2 and 3 for each growth stage) were used for model training, while 48 samples (replicate 1 for each growth stage) were reserved for model validation.

**Figure 2:**
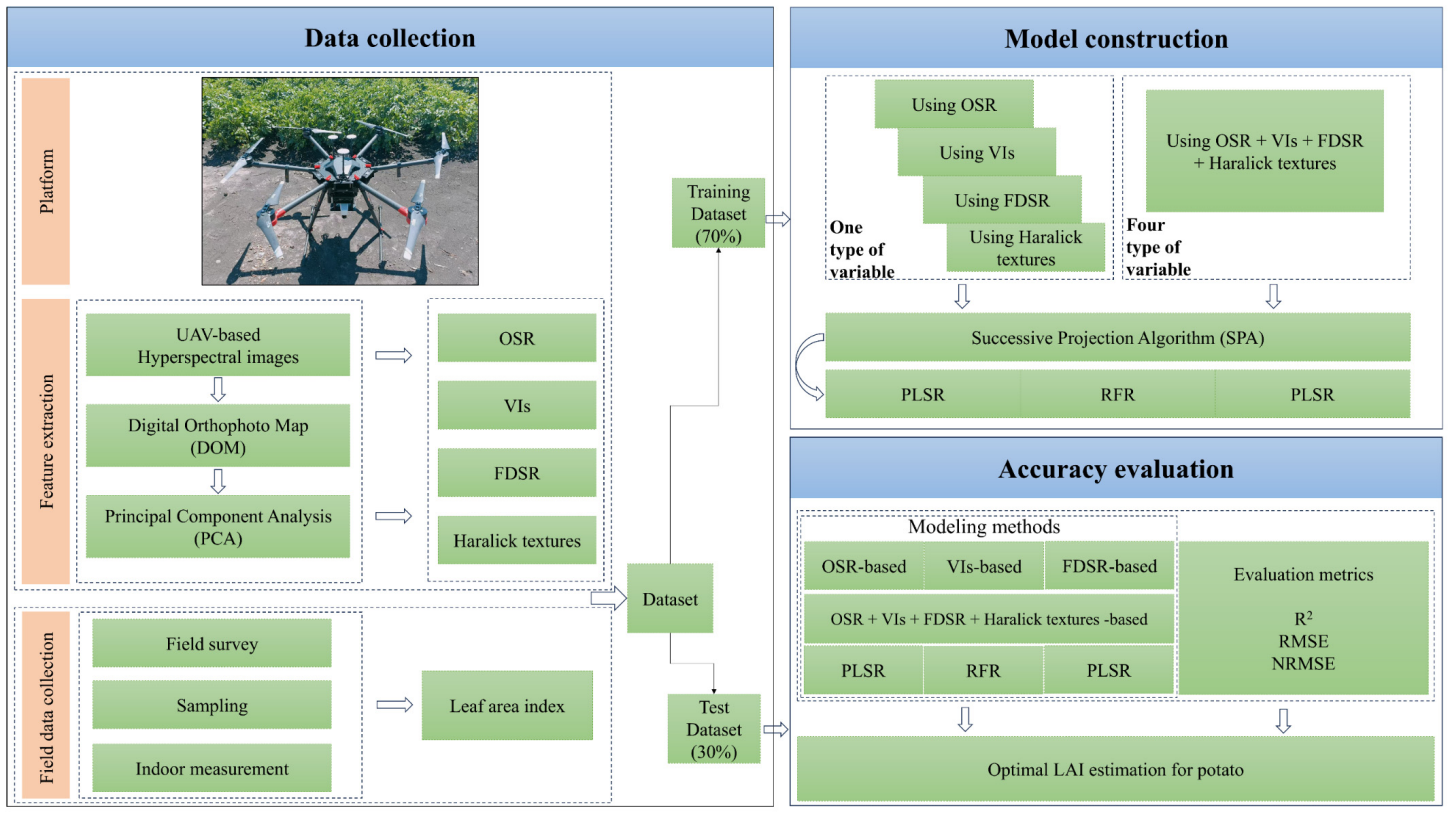
Flowchart of the experimental research.

Our flowchart from different types of spectral and texture extraction to model construction and evaluation in this study is shown in **Figure 2**.

The coefficient of determination (R²), root mean square error (RMSE), and normalized root mean square error (NRMSE) were used to evaluate the accuracy of the models. The corresponding equations are shown in Equations 3–5:


(3) $$R^{2}=\frac{{\left(\sum_{i=1}^{n}y_{i}-\bar{y}\right)}^{2}}{{\left(\sum_{i=1}^{n}x_{i}-\bar{y}\right)}^{2}}$$

(4) $$RMSE=\sqrt{\frac{\sum_{i=1,j=1}^{n}{\left(x_{i}-y_{i}\right)}^{2}}{n}}$$

(5) $$NRMSE=\frac{\sqrt{\frac{\sum_{i=1,j=1}^{n}{\left(x_{i}-y_{j}\right)}^{2}}{n}}}{\bar{y}}$$

Where $x_{i}$ is the measured potato LAI; $y_{i}$ is the estimated potato LAI; $\bar{y}$ is the mean value of the measured potato LAI; n is the number of samples.

## 3 Results and analysis

### 3.1 Analysis of measured LAI

**Figure 3:**
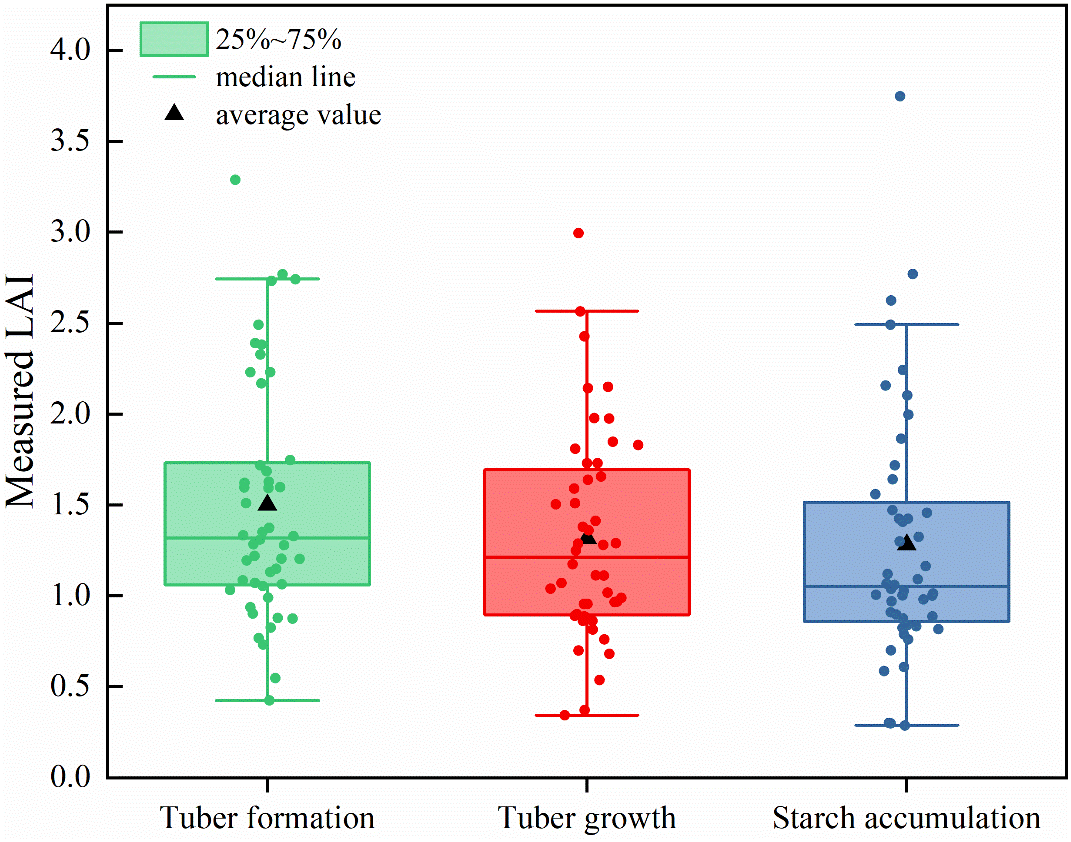
Box line plot of measured leaf area index at each growth stage.

In the field experiment, LAI values of potato at each growth stage in 48 experimental plots were determined. A total of 144 LAI samples were obtained at three growth stages of potato in 2019. The box line plot of potato LAI is shown in **Figure 3**. With the dynamic change of potato growth stage, the mean LAI value first decreased and then levelled off. During the tuber formation stage, the LAI ranged from 0.43 to 3.29, with an average of 1.50. During the tuber growth stage, the LAI ranged from 0.34 to 3.00, with an average of 1.32. During the starch accumulation stage, the LAI varied from 0.29 to 3.75, with an average of 1.28.

### 3.2 Correlation of LAI against hyperspectral image-derived features

**Figure 4:**
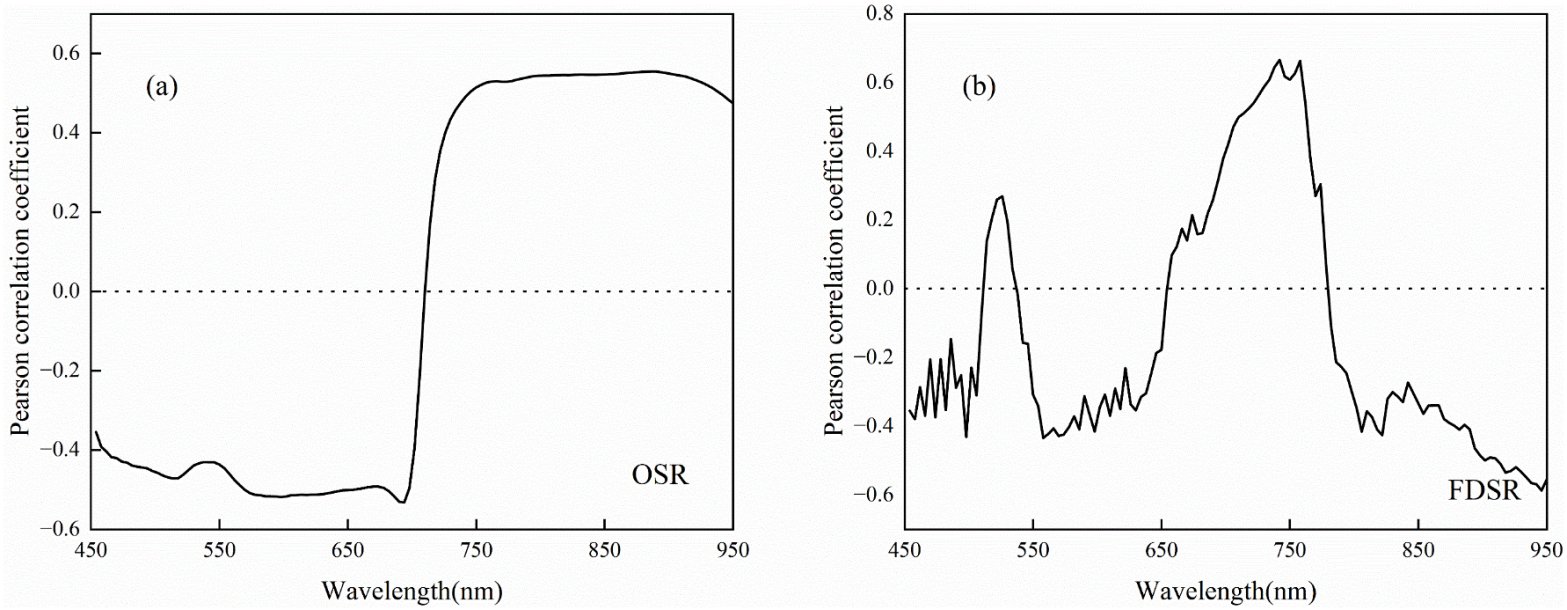
Pearson’s correlation coefficient of LAI with OSR and FOD: **(A)** OSR **(B)** FOD.

**Figure 5:**
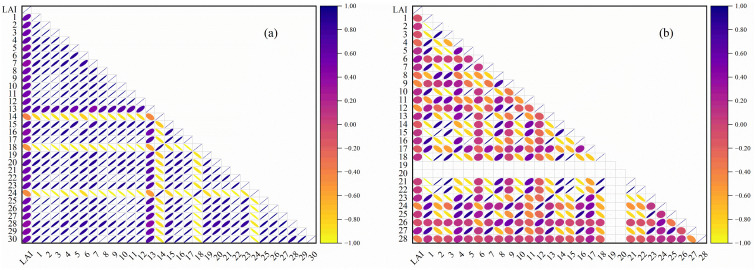
Pearson’s correlation coefficient of LAI with VIs and Haralick textures. **(A)** VIs, **(B)** Haralick textures. The numbers in Panel **(A)** match the Number column in **Table 1**, and the numbers in Panel **(B)** match the Number column in **Table 2**.

The correlation coefficients between different types of characterization variables and the LAI of potatoes across multiple growth stages are presented in **Figures 4**, **5**. LAI exhibited a highly significant correlation with OSR in the wavelength ranges of 454–702 nm and 718–950 nm (*p<* 0.01), with a particularly strong correlation observed at 750–922 nm (**Figure 4A**). The correlation between LAI and FDSR showed greater variability after the first-order derivation of OSR, with highly significant correlations occurring at 454–506 nm, 550–642 nm, 690–774 nm, and 790–950 nm (*p<* 0.01). The highest correlation between LAI and FDSR was noted at 734–758 nm (**Figure 4B**). The VIs selected in this study also demonstrated highly significant correlations with LAI across multiple growth stages of potatoes (**Figure 5A**). Additionally, the extracted Haralick textures, including Inverse Difference Moment, Cluster Shade, Haralick Correlation, Mean, Sum Average, Low Grey-Level Run Emphasis, Short Run High Grey-Level Emphasis, and Long Run Low Grey-Level Emphasis, showed highly significant correlations with LAI (**Figure 5B**).

The variables with the highest correlation to LAI across multiple growth stages of potatoes were 886 nm for OSR (r = 0.55, *p*< 0.01), 742 nm for FDSR (r = 0.67, *p*< 0.01), NDRE for VIs (r = 0.65, *p*< 0.01), and the Mean for Haralick texture (r = -0.50, *p*< 0.01). The mean absolute correlation coefficients of OSR (450–950 nm), FDSR (450–950 nm), VIs, and Haralick texture features with LAI were 0.48, 0.35, 0.53, and 0.17, respectively. These results suggest that VIs may better capture the spatial variability of potato LAI, whereas Haralick texture features may be less effective.

### 3.3 Sensitive feature acquisition using SPA method

The results of filtering five feature datasets using the SPA method are presented in **Table 3**, including OSR, FDSR, VIs, Haralick textures, and their combined features. From a total of 125 OSR features, 125 FDSR features, 30 VIs, 28 textures, and 308 combined features, 10, 7, 8, 10, and 17 features were selected, respectively. This selection indicates that the unselected features were either uninformative or contained redundant information for estimating LAI across multiple growth stages of potatoes.

**Table 3:** Sensitive features were obtained based on different datasets using the SPA method.

Type of features	Features
OSR	462, 474, 514, 542, 594, 674, 694, 714, 798, and 914 nm
FDSR	466, 486, 502, 506, 670, 778, and 946 nm
VIs	EVI, GNDVI, NDRE, NRI, PSRI, PSND, PBI, RVSI
Haralick textures	Inverse difference moment, Inertia, Cluster shade, Mean, Sum of squares: variance, Sum average, Difference entropy, Information measures of correlation IC1, Low grey-level run emphasis, Long run low grey-level emphasis
All	650, 710, 886, and 922 nm (OSR); 478, 530, 586, 630, 702, 738, and 770 nm (FDSR); GNDVI, NDRE, TCARI; Inertia, Cluster shade, Short run high grey-level emphasis

Although the selected wavelengths for LAI estimation using OSR and FDSR differ, they share a common characteristic: these wavelengths span the visible to near-infrared regions and exhibit a high correlation with LAI. Typically, VIs containing near-infrared or red-edge wavelengths are chosen for LAI estimation. Three features were selected from simple textures, five from advanced textures, and two from higher-order textures; however, not all of these features achieved a high correlation with LAI. For instance, features such as Inertia, Sum of squares: variance, Difference entropy, and Information measures of correlation IC1 did not fully reach the highly significant correlation level with LAI. This suggests that the SPA method helps to prevent the omission of features that significantly contribute to LAI estimation. In total, four OSR, seven FDSR, three VIs, and three textures were selected from the combined features, all of which remained highly sensitive to LAI, except for Inertia, which did not achieve a highly significant correlation. These findings indicate that the integration of different types of features is crucial for accurately estimating LAI across multiple growth stages in potatoes.

### 3.4 Estimation of LAI in multiple growth stages of potato

**Figure 6:**
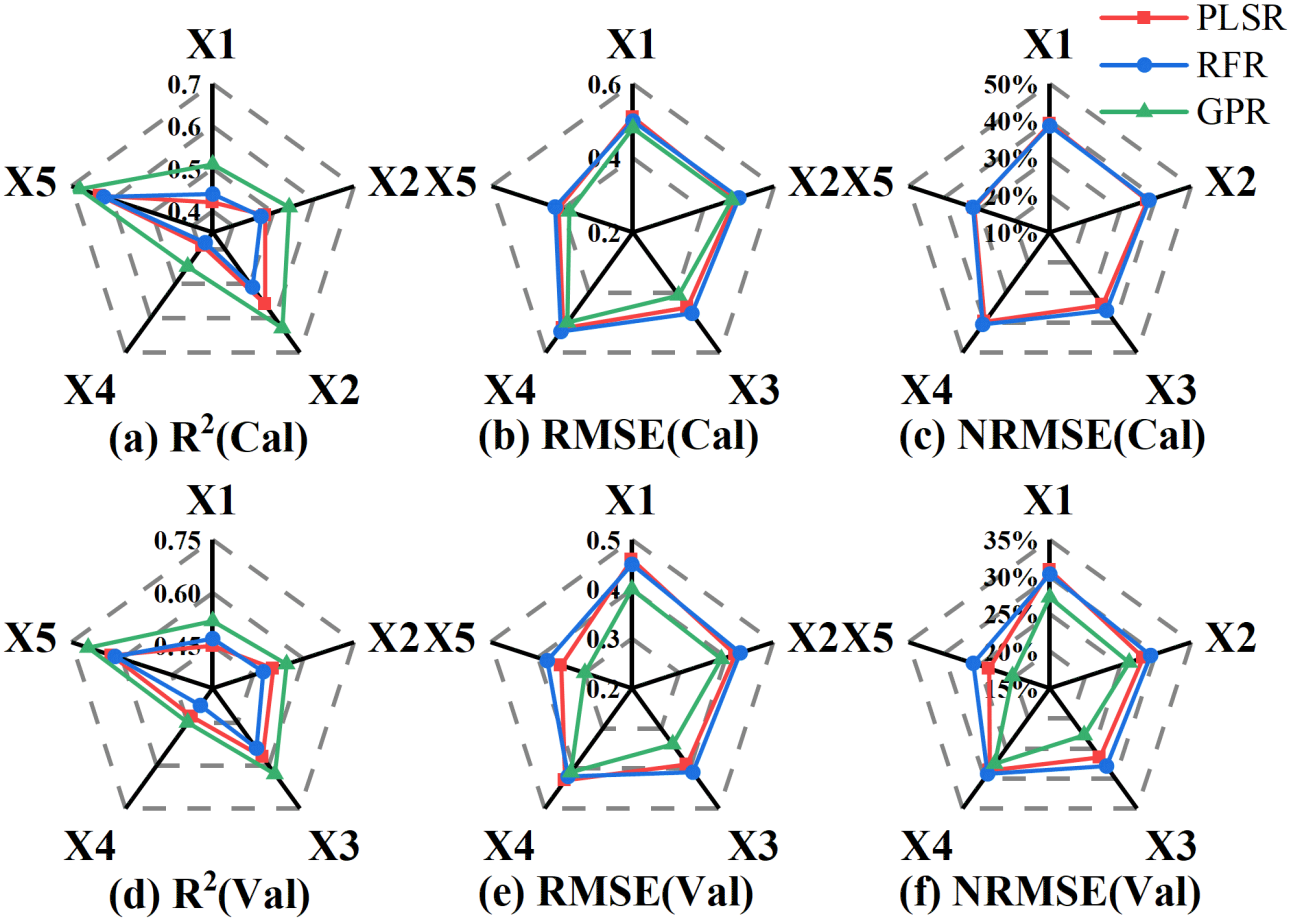
The Accuracy of LAI estimation based on different types of variables. **(A)** R^2^(Cal), **(B)** RMSE(Cal), **(C)** NRMSE(Cal), **(D)** R^2^(Val), **(E)** RMSE(Val), **(F)** NRMSE(Val). X1: OSR, X2: FDSR, X3: VIs, X4: Haralick textures and X5: All features.

The calibration and validation results obtained using PLSR, RFR, and GPR based on the variables screened in **Table 3** are presented in **Figure 6**. X1, X2, X3, X4 and X5 in **Figure 6** represent OSR, FDSR, VIs, Haralick textures and All. Among the models, the LAI estimation model constructed using the GPR method demonstrated higher fitting accuracy and lower error compared to the others. When using the same regression method, LAI estimation based on different types of spectral information was more accurate than that based on Haralick texture features, with the best results achieved using the GPR method with VIs (Calibration: *R*² = 0.63, RMSE = 0.41, NRMSE = 31.32%; Validation: *R*² = 0.63, RMSE = 0.34, NRMSE = 22.84%). Although using Haralick texture features alone to estimate LAI across multiple growth stages of potatoes is not ideal, combining texture features with different forms of spectral information can enhance the accuracy of LAI estimation, suggesting that Haralick texture can compensate for the limitations of spectral information. Compared to the PLSR and RFR methods, the GPR method was the most accurate for estimating multi-stage LAI in potatoes using 17 features that included both spectral and texture information (Calibration: *R*² = 0.68, RMSE = 0.38, NRMSE = 29.04%; Validation: *R*² = 0.70, RMSE = 0.30, NRMSE = 20.28%).

**Figure 7:**
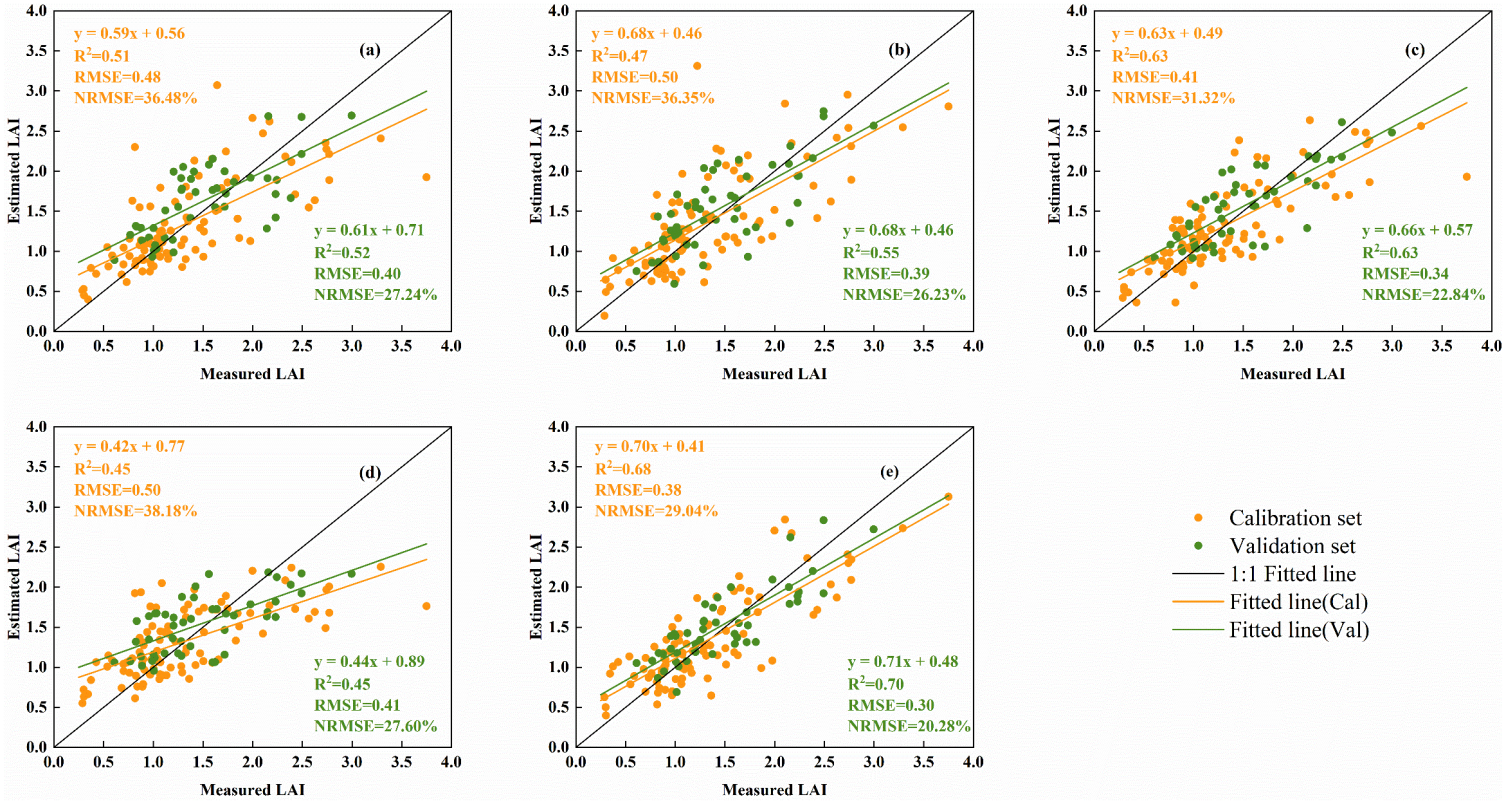
Fitting relationship between measured and estimated LAI based on different types of spectral information and Haralick textures. **(A)** OSR, **(B)** FDSR, **(C)** VIs, **(D)** Haralick textures, and **(E)** spectral information combined with texture features.

The fitted relationship between the estimated and measured LAI values of potatoes across multiple growth stages, obtained using the best regression method based on four types of spectral information, one type of texture, and a combination of spectral and texture features, is illustrated in **Figure 7**. The results indicate the following: (1) Estimating LAI using only spectral information or Haralick textures yields significant errors, particularly with Haralick textures, leading to low model accuracy (*R*² = 0.45, RMSE = 0.41, NRMSE = 27.60%). (2) Among the spectral information, VIs demonstrated the best estimation performance (*R*² = 0.63, RMSE = 0.34, NRMSE = 22.84%). (3) The combination of spectral information and texture features reduced the error in LAI estimation across multiple growth stages and enhanced the accuracy of the model. Notably, when LAI was greater than 2, the estimates based on the fused sensitive features were closer to the 1:1 line with the measured values (*R*² = 0.70, RMSE = 0.30, NRMSE = 20.28%), suggesting that integrating multiple types of features can mitigate the inaccuracies associated with using a single type of feature for LAI estimation.

### 3.5 Applicability of estimated LAI for different growth stages

**Figure 8:**
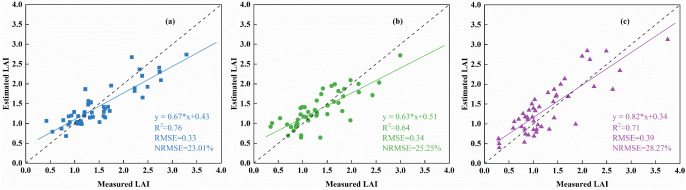
Applicability of estimated LAI for different growth stages based on the optimal model. **(A)** Tuber formation **(B)** Tuber growth **(C)** Starch accumulation.

The study combines three types of spectra and Haralick textures with the GPR machine learning method to estimate LAI in two potato varieties. **Figure 8** illustrates the effect of this estimation to assess the model’s applicability across different growth stages. The results showed that the performance of the method proposed in this study differed significantly in the estimation of LAI for the three growth stages, as evidenced by the poorer performance of the model in terms of accuracy in tuber growth (**Figure 8B**). This is due to the high canopy cover of potato in tuber growth compared to the other two growth stages. Spectral information saturation is more severe and textural information contribution is reduced. In contrast, the accuracy of the model was satisfactory in all three growth stages, although it still varied, so the method of combining different types of spectral information with Haralick textures still has potential for predicting potato LAI in different growth stages.

### 3.6 Mapping potato LAI

**Figure 9:**
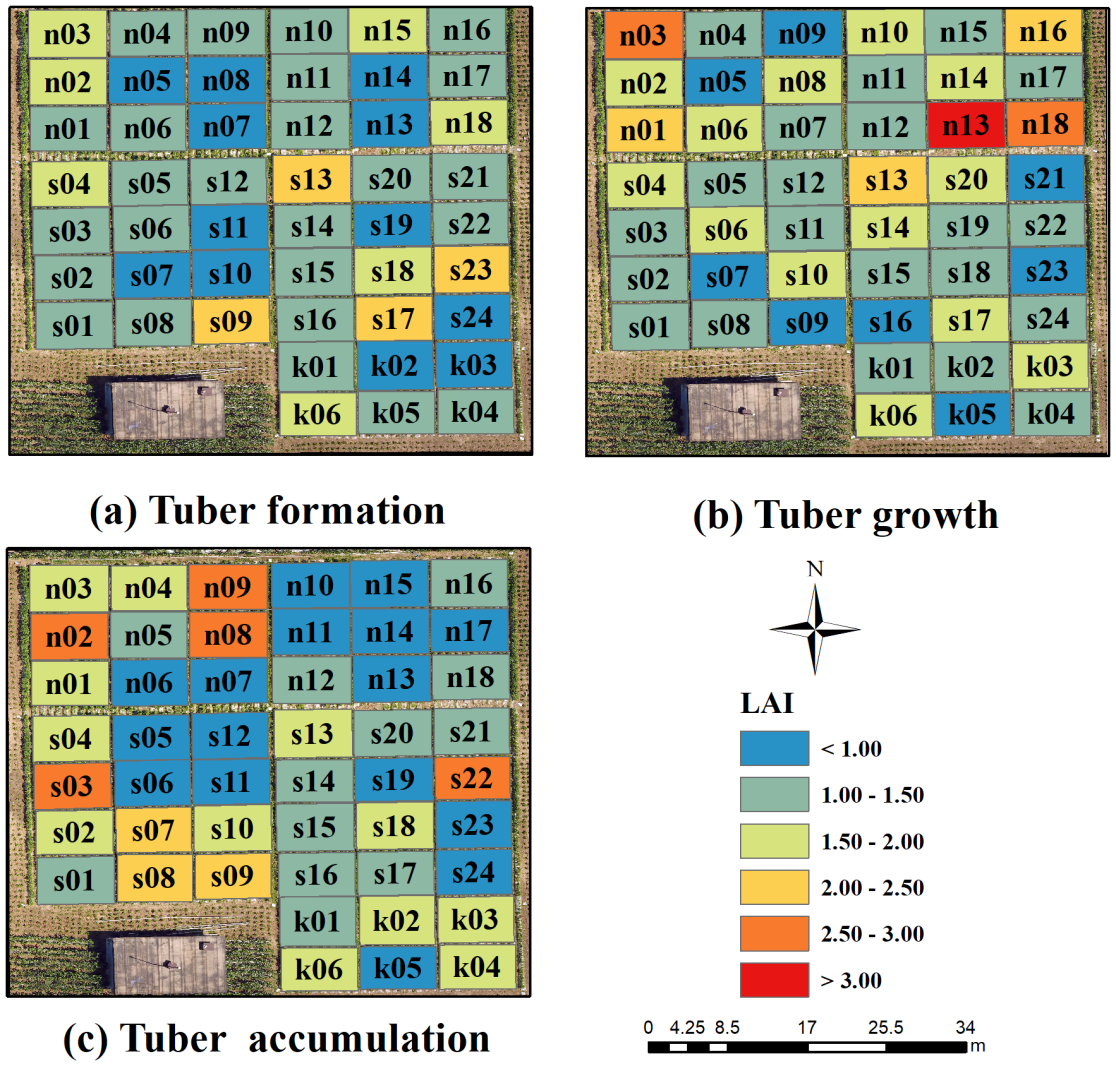
Spatial distribution of LAI in potato at different growth stages. **(A)** Tuber formation, **(B)** Tuber growth, **(C)** Tuber accumulation.

The optimal estimation model of potato LAI for multiple growth stages was constructed based on the three spectral information and Haralick textures, and the model was used to generate the spatial distribution map of potato LAI in the study area, as shown in **Figure 9**. This method helps to provide a more comprehensive understanding of the growth conditions and spatial distribution pattern in the study area. Analyzing LAI spatial distribution maps aids in assessing plant water use efficiency, optimizing irrigation and fertilizer strategies, and enhancing overall water and fertilizer efficiency. Additionally, it supports early detection of pests and diseases, minimizing yield loss, and serves as a preliminary tool for predicting crop yields, ultimately improving agricultural production and management.

## 4 Discussion

Miniature UAV-imaging hyperspectral systems not only acquire spectral reflectance information of the crop canopy but also capture image data of the canopy structure, enabling rapid and non-destructive monitoring of crop LAI (Yan et al., 2019; Verma et al., 2022). Estimating crop LAI across multiple growth stages using only the optical information of the crop canopy is challenging, as the performance of optical data can be inconsistent due to the influence of phenology and crop variety. Given the variations in potato canopy structure and the advantages of hyperspectral imaging, this study explored the applicability and potential of combining Haralick texture features with canopy spectral information for LAI estimation.

### 4.1 Response of spectral and texture features to LAI

The spectral reflectance of the potato canopy reflects the crop’s physiological and geometrical characteristics, while VIs enhance the contrast between crop and soil, allowing for the quantitative description of crop growth. This spectral information is frequently used to estimate crop growth parameters such as LAI (Kayad et al., 2022). The three types of spectra used in this study varied significantly in their sensitivity to LAI across multiple growth stages of potatoes (**Figures 4A**, **B**, **5A**). After differential transformation, FDSR reduced the effects of soil background and overlapping signal interference compared to OSR, thereby enhancing the sensitivity of spectral signals to LAI across different growth stages (**Figures 4A, B**). This finding is consistent with the results obtained by Zhang et al. (2022a) using differentially transformed spectra to estimate the LAI of rice. The VIs selected in this study exhibited higher sensitivity to LAI across multiple growth stages than OSR and FDSR. This increased sensitivity is due to the combination of multiple bands, which can mitigate the interference of external environmental signals on LAI response (**Figure 5A**). However, previous studies by Liu et al. (2022a) and Shi et al. (2022) have shown that the sensitivity of VIs to LAI across different growth stages can be affected by climatic variations and varietal differences, potentially limiting the accuracy of LAI estimation models. Spatial information such as texture of high-resolution UAV remote sensing data correlates with the spatial structure of the crop, reflecting more effectively the shading and internal structural information of the crop canopy. Given the importance of texture information in describing changes in canopy structure, this study attempted to use Haralick texture features to estimate LAI. The application of Haralick texture in the estimation of crop physicochemical parameters has not been extensively investigated. It remains unclear whether the sensitive bands of texture features align with the spectral bands. To address this, we employed the PCA downscaling method, which preserves the majority (>90%) of the image information and offers a robust foundation for subsequent studies. However, compared to canopy spectral information, the simple, advanced, and higher order textures did not effectively capture changes in LAI across multiple growth stages due to the low sensitivity of Haralick texture to potato LAI (**Figure 5B**), which is similar to the previous work of Qiao et al. (2024). The extraction of texture features from an image is often affected by a variety of factors, such as noise in the image and changes in lighting conditions, which may interfere with the stability of the texture features. This limitation may be related to the size of the window used for texture extraction and the calculation method employed. Consequently, we conclude that Haralick texture alone does not adequately reflect changes in LAI across multiple growth stages of potatoes.

### 4.2 Optimization of model parameters

To reduce the number of input parameters for the LAI estimation model and minimize errors due to subjective variable selection, the feature variables obtained using the SPA algorithm are presented in **Table 3**. For both OSR and FDSR, the characteristic wavelengths identified after SPA screening, although not identical, fall within the visible to near-infrared regions. Since LAI is determined by plant leaves, and the absorption and reflection of pigments within these leaves, along with scattering between leaf tissues, influence the crop canopy spectra across the visible to near-infrared ranges, the differences in the sensitive spectral positions of various transformed spectral data and LAI are expected to be minimal (Zhou et al., 2022). The eight VIs selected through screening all include red-edge or near-infrared wavelengths, as these spectral regions are more responsive to changes in LAI (**Figures 2A, B**), a finding consistent with studies by Tao et al. (2020) and Liu et al. (2022a) in monitoring winter wheat LAI. The fact that not all of the 10 variables selected from the 28 Haralick textures achieved a highly significant correlation with potato LAI suggests that the variables identified by the SPA algorithm are objectively determined, allowing for the retention of those that significantly contribute to LAI estimation and thereby avoiding the large estimation errors that can result from subjective variable selection. To evaluate the performance of combining spectral and texture information for LAI estimation, four OSR, seven FDSR, three VIs, and three textures were selected from 308 combined features, underscoring the importance of feature fusion in accurately estimating LAI in potatoes across multiple growth stages (**Table 3**).

### 4.3 Performance evaluation of LAI estimation model

Compared to traditional algorithms, machine learning regression models can make effective use of data when dealing with complex data and obtain higher model prediction accuracy. The modeling and validation results obtained using the GPR method for the variables identified in **Table 3** were superior to those achieved with the PLSR and RFR methods. This finding aligns with the results reported by Caballero et al. (2022) for estimating LAI in winter wheat. The superior performance of GPR may be attributed to its ability to effectively handle nonlinear relationships between independent and dependent variables, as well as its suitability for small sample datasets (Upreti et al., 2019; Zhang et al., 2022b). In this study, the modeling and validation datasets were small, and the spectral and texture information extracted from UAV hyperspectral imagery likely exhibited a stronger nonlinear relationship with LAI, making GPR a fitting choice for constructing the LAI estimation model. Analyzing the results in **Table 3**, the accuracy of LAI estimation across multiple growth stages of potatoes was higher when using the three forms of spectral information compared to Haralick texture alone. This is consistent with the findings of Zhang et al. (2022b) in estimating LAI for maize, where the grey-scale covariance matrix texture was less effective than crop canopy spectral information. Among the spectral information types, the order of performance in estimating LAI was VIs, FDSR, and OSR, which corresponds to the sensitivity of these spectral types to LAI (**Figures 4A**, **B**, **5A**). Although Haralick texture alone was not effective for estimating potato LAI (**Table 3**, **Figure 3**), combining it with crop canopy spectral features improved the accuracy of LAI estimation, consistent with Zhou et al (Zhou et al., 2022), who combined wavelet texture with spectral features for estimating rice LAI. Further illustrating that different types of feature coupling provide unique and complementary information with great potential in crop monitoring (Ochiai et al., 2024). Yu et al. (2023) also demonstrated that combining different types of features can improve the prediction performance of LAI in potato. This study highlights the limitations of Haralick texture, but also demonstrates its value as auxiliary information when combined with spectral features for LAI estimation. The combination of spectral and Haralick texture information in this study explained 69% of the variability in LAI across multiple growth stages in potatoes, which is lower than the 85% of spatial variability in maize LAI explained by Zhang et al. (2022b) using fusion information. This discrepancy may be due to differences in crop variety and canopy structure.

### 4.4 Advantages and limitations of research

Haralick texture differs from spectral information in response to potato canopy structure, and their complementary information helps to improve LAI estimation accuracy. This complementary information can enhance the accuracy of LAI estimation. In this study, models for LAI estimation that integrated both spectral and texture data outperformed those relying on a single type of data. Thus, combining spectral information with Haralick textures offers valuable insights for LAI estimation across various growth stages of potatoes. The study derived the spatial and temporal distribution of potato LAI at the field scale using the optimal estimation model (**Figure 9**), facilitating the assessment of the model’s potential for practical field applications. Despite achieving satisfactory results, some limitations remain. Future research should consider incorporating additional crop structural characteristics, such as vegetation cover, plant height, and volume, to improve model construction. This study compared the potential of three machine learning methods for UAV estimation of LAI in wheat, but found that deep learning performed better for potato biomass prediction (Liu et al., 2024c), suggesting that deep learning has greater potential for application in the field of UAV spectral monitoring of crop growth. Therefore, future research will deeply explore the application of methods such as deep learning in this field. In addition, since the UAV and ground data used in this study were limited to a single location, within one year, and two varieties, we will further explore the impact of multi-year and multi-variety potato data on improving the performance of Haralick texture features. Future work will focus on different varieties and multiple years of data to analyze the robustness of canopy spectra and Haralick texture features in estimating potato LAI.

## 5 Conclusions

To accurately and timely estimate potato LAI, this study utilized UAV hyperspectral image data to extract three types of spectral information and Haralick textures, and analyzed their combined ability to estimate potato LAI. The effectiveness of this method was validated using plot test data, yielding the following results:

1. Using the SPA feature selection method, 17 sensitive features were identified as significant contributors to potato LAI, including four OSR wavelengths (650, 710, 886, and 922 nm), seven FDSR wavelengths (478, 530, 586, 630, 702, 738, and 770 nm), three VIs (GNDVI, NDRE, TCARI), and three Haralick textures (Inertia, Cluster shade, Short run high grey-level emphasis).
2. Among the single-type feature models, VIs demonstrated the best performance. In contrast, the regression performance of Haralick textures was less effective when used independently.
3. The accuracy of potato LAI estimation improved when combining features as input parameters, achieving an *R*² of 0.70 and an RMSE of 0.30. This model outperformed the optimal VIs model based on univariate parameters (*R*² = 0.63, RMSE = 0.34), suggesting that the combination of spectral information and Haralick textures provides better accuracy for LAI estimation and offers a feasible approach for monitoring potato growth.

Combining different types of spectral information and Haralick textures from UAV hyperspectral imagery can improve the estimation of LAI for potatoes with multiple growth stages to some extent compared to using a single type of feature. However, UAV multispectral data can provide higher spatial resolution and may be more suitable for texture feature extraction. Therefore, an attempt was made to validate the feasibility of this study’s method using low-cost UAV multispectral in future studies.

## Data Availability

The original contributions presented in the study are included in the article/supplementary material, further inquiries can be directed to the corresponding author/s.
